# Exercise and colorectal cancer: prevention and molecular mechanisms

**DOI:** 10.1186/s12935-022-02670-3

**Published:** 2022-08-09

**Authors:** Ramin Amirsasan, Maryam Akbarzadeh, Shabnam Akbarzadeh

**Affiliations:** 1 Department of Exercise Physiology, Faculty of Physical Education and Sport Sciences, University of Tabriz, Tabriz, Iran; 2 Department of Biology, Faculty of Natural Sciences, University of Tabriz, Tabriz, Iran

**Keywords:** Exercise, Physical activity, Colorectal cancer, Prevention, Mechanisms

## Abstract

Exercise and physical activity have been shown to be strongly associated with a decreased incidence rate of various chronic diseases especially numerous human malignancies. A huge number of clinical trials and meta-analysis have demonstrated that exercise is significantly effective in lowering the risk of colorectal cancer. In addition, it is suggested as an effective therapeutic modality against this cancer type. Therefore, in this review, we will review comprehensibly the effects of exercise in preventing, treating, and alleviating the adverse effects of conventional therapeutic options in colorectal cancer. Moreover, the possible mechanisms underlying the positive effects of exercise and physical activity in colorectal cancer, including regulation of inflammation, apoptosis, growth factor axis, immunity, epigenetic, etc. will be also discussed.

## Key points

- Exercise is an effective post-treatment management program in colorectal cancer survivals
- Exercise improves muscle strength, cardiorespiratory fitness, emotional distress, physical activity, fatigue, and sleep quality in colorectal patients undergoing chemotherapy
- Targeting and modulating insulin-like growth factor (IGF) system, inflammation, apoptosis, immunity, epigenetic, Leptin and Ghrelin, and signaling pathways are major underlying mechanisms for preventive effects of exercise in colorectal cancer

## Introduction

Colorectal cancer (CRC) is one the most frequently occurred cancer types among various populations. Its incidence rate is increasing every day, such that it is estimated that the number of CRC survival will grow to 2.5 million in 2035 [1, 2]. There is a great variation in the incidence patterns of CRC among world regions. More importantly, in the past few decades, multiple factors such as economic development, inappropriate lifestyle and dietary habits, some important cases of them include, consuming high amounts of red/processed meats, fats, sugary foods, refined grains, alcoholic beverages, and low amounts of dietary fiber, vegetables, and fruits result in the considerable increase in the CRC incidence and numbers of patients. Smoking, physical inactivity, hence overweight, and obesity are other factors affecting he incidence rate of CRC [3, 4]. Therefore, physical activity, hormone therapy in postmenopausal women, aspirin use, fruit consumption, and vegetable consumption are associated with decreased risk of CRC [5]. Oxidative stress, inflammation, and metabolic dysfunction are considered as three important and well-studied underlying mechanisms for the initiation and development of CRC [6, 7]. However, due to the high complexity of CRC development, the participation of various genetic and environmental factors and mechanistic pathways in this process, CRC etiology is still unknown and needs further studies [8, 9].

There are various therapeutic strategies such ad surgery, chemotherapy, and radiotherapy for combating CRC [10]. However, their limitations such as severe side effects, tumor recurrence, and developing resistance, more importantly, the presence of metastatic disease at the time of diagnosis, result in urgent need for developing novel therapeutic modalities that effectively remove tumors and increase patient's survival and prognosis [10]. In recent years, an accumulating number of studies have focused on the preventive and therapeutic effects of exercise and physical training, as one of the major lifestyle factors in numerous human malignancies [11]. Completed clinical trials and meta-analysis have reported that physical activity and exercise are significantly effective in lowering the risk of various human malignancies such as breast, proximal and distal colon, gastroesophageal, endometrial, ovarian, prostate, renal, pancreatic, and lung cancer [12, 13]. In addition to preventive effects, physical activity also has been reported to be an effective therapeutic modality against colorectal cancer (Table 1). This review tries to have a comprehensive and up-to-date overview of the exercise and physical training as a preventive and thematic strategy against colorectal cancer, as well as the underlying molecular mechanisms with special attention to animal and human studies, as well as clinical trials.

**Table 1:** Positive effects of exercise training and physical activity in colorectal cancer patients and animal models

Study	Sample size	Conditions	Type of exercise	Major finding	Refs.
Colbert (2001)	29,133 men	50–69 years old	occupational and leisure-time activities	findings provide further evidence of an inverse association between physical activity and colon cancer The relationship appeared to be stronger in the distal colon, rather than the proximal colon, and a similar association was seen for rectal cancer	[14]
Thune (2001)	40,674 patients	N/A	Occupational physical activity Leisure time physical activity	A dose–response effect of physical activity on colon cancer risk was especially observed	[15]
Chao (2004)	940 colon and 390 rectal cancer patients	Mean age of 63 years old	Recreational Physical Activity	Increasing amounts of time spent at recreational physical activity are associated with substantially lower risk of colon cancer Recreational physical activity is associated with lower risk of rectal cancer in older men and women	[16]
Calton (2006)	31,783 women	Mean age of 61 years old	Daily physical activity	Data do not support the hypothesis that physical activity is related to a lower incidence of colon cancer	[17]
Mai (2007)	120,147 participants	22–84 years old	Lifetime recreational physical activity	Lifetime recreational physical activity may protect against colon cancer among postmenopausal women who have never used hormone therapy Among hormone therapy users, who have lower risk of colon cancer, recreational physical activity does not seem to provide any additional benefit With declining rates of hormone therapy use, physical activity offers one possible means for reducing women's colon cancer risk	[18]
Coups (2008)	1932 respondents to the Health Information National Trends Survey	18–70 years old	Moderate-intensity activities	There is poor awareness among U.S. adults of the role that physical activity plays in preventing colon cancer	[19]
Wolin (2010)	158,253 participants	N/A	Regular long-term physical activity	Regular long-term physical activity was associated with a lower risk of colon cancer mortality	[20]
Boyle (2011)	870 cases	40–71 years old	Recreational Physical	Physical activity may have a greater effect on the risk of distal colon cancer than proximal colon cancer Vigorous physical activity is required to reduce colorectal cancer risk	[21]
Sanchez (2012)	548 patients	Mean age of 58 years old BMI of 27	Exercised for at least one hour per week	Exercise was an independent negative predictor for the presence of adenomas anywhere in the colon Patients who reported exercising one or more hours weekly had a lower prevalence of any polyps	[22]
Kuiper (2012)	1,339 participants	Mean age of 65 years	Recreational physical activity	Patients reporting activity levels ofC18 MET-h/week had significantly lower colorectal cancer-specific mortality	[23]
Weijenberg (2013)	120,852 participants	55–69 years old	Occupational physical activity	Regular long-term physical activity and fewer sitting hours may protect against colon cancer, particularly distal colon cancer	[24]
Moore (2016)	1.44 million participants	Median [range] age, 59 [19–98] years old	Leisure-time physical activity of a moderate to vigorous intensity	Leisure-time physical activity was associated with lower risks of colon cancer	[25]
Aleksandrova (2017)	519 978 participants	25–70 years old	High physical activity	Promoting physical activity, particularly outdoors could represent a promising strategy for colon cancer prevention	[26]
Mahmood (2018)	23,586 patients	27 to 76 years old	Recreational activity	Recreational activity was associated with reduced CRC risk. A non-significant, inverse association was observed for occupational activity, whereas no association was found for transport or household domains	[27]
Animal models					
Baltgalvis (2009)	48 Apc(Min/ +) mice	Four-week-old male mice	Regular moderate-intensity treadmill exercise (18 m/min, 60 min/d, 6 d/wk)	The induction of adiposity, inflammation, and immunosuppression by the Western-style diet may compromise the beneficial effect of moderate-intensity exercise on the intestinal polyp burden in Apc(Min/ +) mice	[28]
Kelly (2017)	N/A	Eight-weeks of age male mice	Voluntary running wheel access	The results indicate that voluntary exercise should be used as a preventative measure to reduce risk for environmentally induced CRC with the realization that the extent of protection may depend on genetic background	[29]

## Exercise and cancer

There are various mechanisms responsible for the preventive and therapeutic effects of exercise and physical activity on cancer [30]. One of the most significant mechanisms is the modulation of proliferative signaling pathways. Disruption of proliferative signaling pathways decreases the likelihood that cellular malignant transformation will occur [30, 31]. Studies in animal models of multiple cancer types have shown decreased levels of various mitogenic hormones such as insulin growth factor-1 (IGF-1) and their downstream signaling pathways with potent proliferative and anti-apoptotic effects, including Ras-mitogen-activated protein kinase (MAPK), phosphoinositide 3-kinase (PI3K)-Akt and Janus kinase (JAK)/signal transducer and activator of transcription (STAT) signaling transductions prevent cancer initiation and progression [32–35]. More interestingly, exercise also results in alteration of serum factors, which leads to the upregulation of p53 and activation of downstream anticancer signaling [36]. Another major mechanism in developing cancers is the inactivation of tumor suppressor genes [37]. Exercise is reported to upregulate the expression levels of important tumor suppressor genes, including p53, p21, insulin-like growth factor-binding protein (IGFBP) -3, programmed cell death (PDCD)-4, and phosphatase and tensin homolog (PTEN) [37]. In the murine model of mammary carcinogenesis, exercise resulted in decreased levels of hyper-phosphorylated retinoblastoma protein [38, 39]. Exercise is also reported to downregulate miR-21 and the anti-apoptotic protein Bcl-2 and increase the expression levels of the tumor suppressor PDCD4 in an animal model of breast cancer [40]. Resistance to apoptosis along with disruption in the proliferative pathway is common events in tumor formation [41]. In animal models of pancreatic, prostate, skin, and breast cancers, it was found that physical activity and exercise effectively inhibited tumor growth and induced apoptosis through activation of caspase-3 and p53 and inhibition of Bcl-2 [41–45]. Higgins et al. demonstrated that exercise led to significant upregulation of p53, as well as increased expression levels of pro-apoptotic proteins, Bax and Bak, hence delay of lung adenocarcinoma tumor growth [46]. Exercise is indicted to play an active role in modulating the expression levels of angiogenesis-related genes, hence regulating angiogenesis and metastasis processes during cancer progression [47]. Vascular endothelial growth factor (VEGF) and hypoxia-inducible factor-1 alpha (HIF-1a) are two major players of angiogenesis in the tumor microenvironment that are upregulated by exercise in various animal models of cancer [47]. Therefore, exerciser training results in the suppression of invasion and metastasis of cancer cells through normalization of the tumor microenvironment [47–52].

**Fig. 1:**
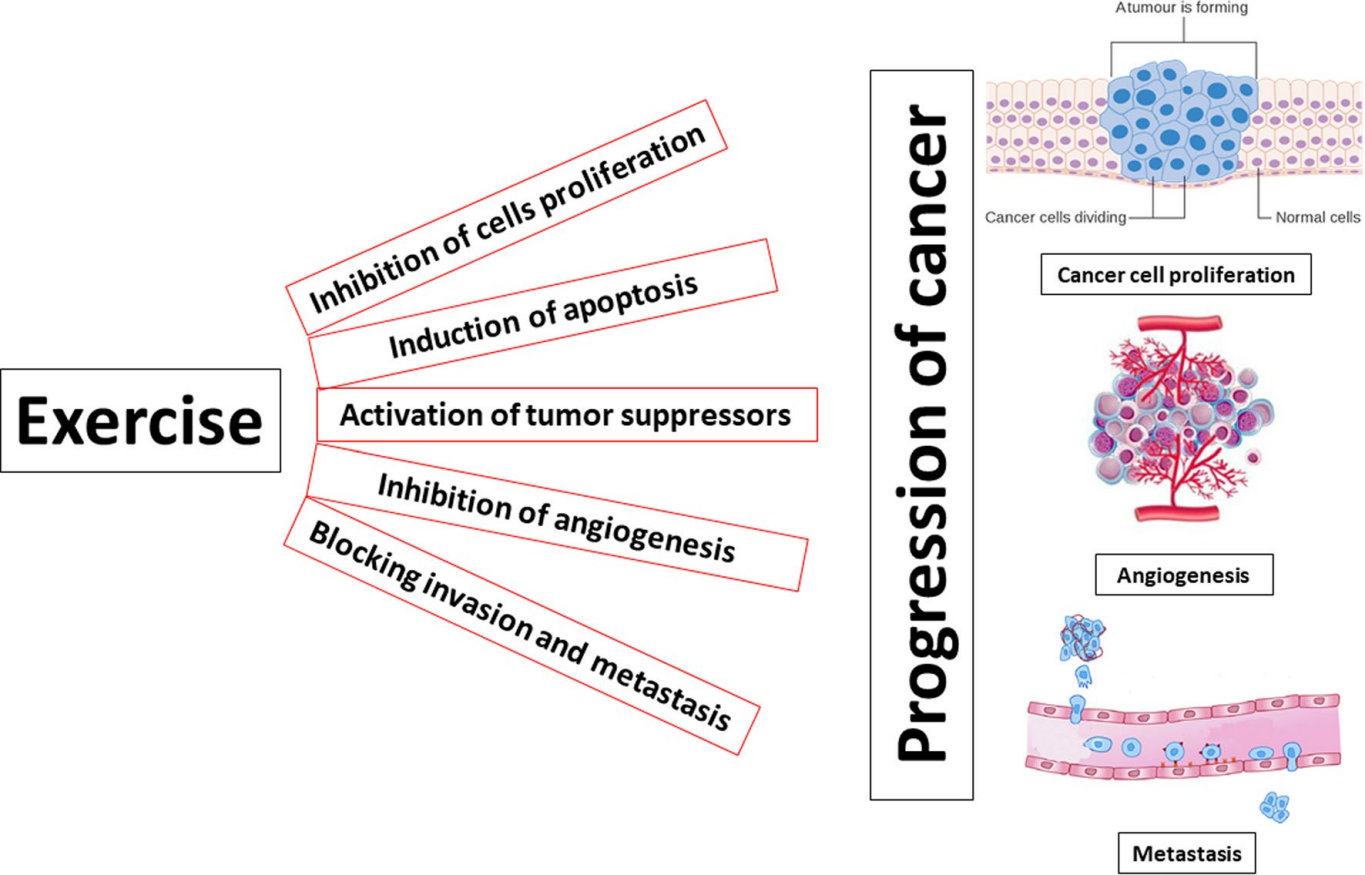
molecular mechanisms underlying therapeutic effects of exercise in cancer

The preventive functions of exercise and physical activity and their effects on reducing the incidence risk of various human malignancies are the most studies areas of cancer treatment [53]. These effects are more prominent in the case of colorectal and breast cancer, such that there are more than one hundred studies only for investigating the effects of exercise on reducing the risk of CRC [54]. An accumulating number of these studies have convincingly reported an approximately 24–40% decrease in the CRC risk in physically active individuals in comparison to the least active [54–60]. Despite extensive investigation, however, there are still some unrevealed aspects of the association between physical activity and reduced risk of colorectal cancer [21]. Two important issues in this field are the timing and intensity of physical activity in relation to CRC risk, which will comprehensively discuss in the following paragraphs (Fig. 1).

### Timing of exercise

Results from various investigations about the appropriate age periods, in which physical activity exerts more potent preventive effects in CRC patients, have found that being active and exercise in any age periods of life, including 30–39, 40–49, and + 50 years has a strong association with the reduced risk of CRC. In other words, exercise during the 30–50 years of a person's life is more consistently related to reducing risk [16, 61–63]. This finding is mostly concluded from the results of case–control studies, which have used exercise questionnaires that decrease the validity and reliability of related studies. Although it is clear and mostly accepted that 30–50 years is the age period, in which exercise may optimally decrease CRC risk, it is also possible that people can recall the amount of physical activity they performed in this age period more reliably than in other age periods [54]. For example, in a study consisted of 3,240 men and 1,482 women with CRC and healthy controls, the association between physical activity during different ages (15–18 years, 19–29 years, and in the past 10 years) and reduced CRC risk was evaluated and it was found that total physical activity at ages 15–18 and ages 19–29 years was not associated with colon cancer, whereas a decrease in colon cancer risk was observed with increasing levels of total physical activity at ages 35–39 years and increasing levels of total lifetime physical activity [64]. On the other hand, decreased CRC risk is also associated with long-term or lifetime physical activity. This is based on the finding of case–control and cohort studies, which reported the reduced risk for individuals performing consistently high levels of physical activity [54].

### Intensity of exercise

According to the metabolic-equivalent (MET) value of different physical activities, they are classified into three distinct categories, including light (a MET value between 1.6 and 2.9), moderate (a MET value between 3.0 and 5.9) and vigorous (a MET value equal to or greater than 6.0). Activities such as standing and most household chores are considered as light-intensity activities [65]. Walking for exercise, golf, and gardening are moderate-intensity activities and running, swimming, and squash are placed in the vigorous category. The number of epidemiologic studies investigated the physical activity in association with CRC risk, have compared the most active individuals with the least active ones [66]. However, it is suggested that physical activity may result in a 24% risk reduction in men and 23% risk reduction in women. The reason why the findings are less consistent for women is unclear. Because hormone therapy is associated with reduced risk of colon cancer among postmenopausal women, it may mask any beneficial effects of physical activity on colon cancer risk among women with a history of hormone use. In the case of comparing vigorous-intensity activity with moderate-intensity activity, it is found that vigorous-intensity activity confers a greater risk reduction (26%). This conclusion is based on three case–control studies. There is not any cohort study for approving this finding. An increase in insulin sensitivity, downregulation of IGF signaling, decrease in obesity are among the most important and accepted biological mechanisms for preventive effects of vigorous-intensity physical activities [67]. Another possible explanation is that vigorous-intensity physical activity is recalled more reliably than moderate-intensity meaning that vigorous activity is better able to distinguish between highly active and inactive participants [68–70].

### Sedentary behavior

Any activity needing low energy expenditure such as prolonged sitting or watching television or working at a desk is considered sedentary behavior, which is confirmed by an increasing number of studies as an independent risk factor for multiple chronic diseases. In addition, sedentary behaviors have also a close association with an increased incidence of CRC (30%). However, these findings and suggestions are made based on hospital-based case–control studies evaluated occupational activity [71, 72], which have major limitations. In other words, light-intensity activity has also demonstrated to have health benefits such as decreasing CRC incidence [73]. The effect of too much sitting on adiposity, metabolic dysfunction, inflammation, and vitamin D has been proposed to be the pathways through, which sedentary behavior may influence colon cancer risk [71]. In addition, Whitemore et al. reported that saturated fat intakes exceeding 10 g/day, particularly in combination with physical inactivity, could account for 60% of colorectal cancer incidence among Chinese-American men and 40% among Chinese-American women [74].

## Exercise in colorectal cancer survivals

Due to the high importance of post-treatment for cancer survivors, sufficient post-treatment management is considered as one of the most important issues for improving the health and quality of life of survivors [75]. As mentioned before, great advances in the detection and therapeutic strategies for CRC, the number of CRC survivors is sharply increasing, which needs an effective post-treatment management program. Despite the existing multiple post-treatment management programs, there are not sufficiently effective universal guidelines [76]. Major changes in lifestyle such as physical activity and proper dietary habits are two key elements of these programs. Especially, studies have shown reduced physical activity in CRC survivors in comparison to other cancer survivors [77]. Lynch et al. reported that 68% of CRC survivors have physical inactivity after the treatment period [78]. Furthermore, in spite of approved beneficial effects of exercise and physical training, it has been demonstrated that only 23.5% of CRC survivors followed the exercise guidelines [79]. The majority of the barriers to sports participation reported by CRC survivors are similar to those identified for healthy populations (e.g., time, age/agility, distance to travel, cost); however, some of the barriers identified are unique to CRC survivors (e.g., poor bladder control, having an ostomy) [77]. Physical activity and exercise are considered as one of the most important and effective post-treatment managements for CRC survivors, which have been reported to enhance patients' fitness and improve their quality of life [80]. In addition, exercise also decreases the risk of tumor recurrence and developing chronic diseases, including cardiovascular disease and diabetes, hence all-cause mortality in CRC survivors [81, 82]. Improving cardiorespiratory fitness and body composition, are other promising positive effects of exercise for CRC survivors [83]. Moreover, exercise and an appropriate lifestyle alleviate the treatment-induced long-term and severe side effects [84, 85]. For example, Grimmett et al. demonstrated that increase physical activity along with fruit and vegetable intake, and reduce consumption of red/processed meat and alcohol in CRC survivors who had recently completed treatment resulted in the significant improvement in patients quality of life [86]. Other important beneficial impacts of exercise and physical activity in CRC survivors include improving quality of life, lymphedema, functional status, weakness, and muscle strength [87–89]. Table 2 has shown a comprehensive list of researches about outcomes of exercise in CRC survivors, who competed all surgery, chemotherapy and/or radiation.

**Table 2:** The positive effects of exercise in colorectal cancer survivors

Study	Sample size	conditions	Type of exercise	Major finding	Refs.
Ligibel (2012)	237 patients	stage I–III CRC survivors	180 min of moderate-intensity physical activity	Survivors enrolled in a multicenter, telephonebased physical activity intervention increased physical activity and experienced significant improvements in fitness and physical functioning	[90]
Cheville (2013)	66 patients	Stage IV CRC survivors	8 weeks incremental walking and home-based strength training	A home-based exercise program seems capable of improving the mobility, fatigue, and sleep quality of patients with Stage IV lung and colorectal cancer	[91]
Chung (2013)	431 pateints	Mean age of 58 years old	Strenuous, moderate and mild physical activity	Survivorswho were older or received chemotherapy increased their total PA and mild intensity PA after the completion of treatment	[79]
Sellar (2013)	888 pateitns	stage II or III CRC survivors	12-week supervised exercise intervention	Exercise training was found to be feasible and improved many aspects of health related physical fitness in CRC survivors that may be associated with improved quality of life and survival in these individuals	[81]
Courneya et al. (2014)	250 patients	High-risk stage II or stage III CRC survivors	A three-year exercise program	The Colon Health and Life- Long Exercise Change (CHALLENGE) trial was proven to be effective as a randomized controlled trial assessing the effect of an exercise program on disease-free survival	[92]
Grimmett (2015)	29 patients	Patietns over 18 years old who had recently completed treatment within the last 6 months	12 week intervention for increasing physical activity	Meaningful improvement in quality of life was observed	[86]
Husson (2015)	6446 patients	Mean age of 71 years, stage I–III CRC survivors	Physical activities including walking, bicycling, gardening, housekeeping, and sports	Pateitns with PA have higher on the global quality of life, physical, role, cognitive, emotional, and social functioning over time Finding underlines the importance to focus upon training in survivorship care and strategies to get inactive cancer survivors physically active	[93]
Courneya (2016)	273 patients	High-risk stage II and III colon cancer survivors	Gradually increase recreational PA from baseline by 10 MET hours/week	The behavior change intervention produced a substantial increase in selfreported recreational PA that met the feasibility criterion for trial continuation, resulted in objective fitness improvements, and is consistent with the amount of PA associated with improved colon cancer outcomes in observational studies	[94]
Grote (2016)	11 patients	N/A	Blended aerobic and resistance training (CART) three days per week for 13 weeks	The study revealed a close relationship between CART and cancer survivors’ cardiometabolic health After 13 weeks of training, participants experienced an average decrease in waist circumference Decrease in waist circumference was associated with a decrease in CRP	[95]
Fisher et al. (2016)	495 patients	Patients who were between 6 months and 5 years post-diagnosis with non-metastasised disease	Post-diagnosis PA	The findings indicated the benefits of PA following cancer treatment, while also identifying barriers to effective implementation	[96]
Cantarero-Villanueva (2017)	46 patients	older than 18 years, stage II–III CRC survivors	Lumbopelvic Exercise Program	PA improves musculoskeletal conditions in the lumbopelvic area of CRC patients, specifically in terms of pain and internal oblique thickness	[97]
Chen et al. (2017)	116 pateitns	Elderly patients prepared for CRC surgery	Four-week trimodal rehabilitation program	The results indicated that the trimodal rehabilitation program had a positive effect on levels of PA, as well as on functional walking ability The results indicate the potential to improve PA and physical function among elderly cancer patients	[98]
Forbes et al. (2017)	95 patients	N/A	Internet-delivered, distance-based PA	The Internet-based program was proven to have a negative impact on cancer survivors’ motivation	[99]
Brown (2016 and 2017, 2018)	39 patients	Stage I–III CRC survivors	150 min/wk of aerobic exercise (low dose) and 300 min/wk of aerobic exercise (high dose) for 6 months	Aerobic exercise reduces visceral adipose tissue in dose–response fashion among patients with stage I–III CRC Visceral adipose tissue may be a mechanism through which exercise reduces the risk of disease recurrence among CRC survivors	[100–102]
Devin (2016 and 2018)	47 patients	Post-treatment CRC survivors	4 weeks of moderate intensity exercise (MIE) and high intensity exercise (HIE) training	In response to short-term training, HIE is a safe, feasible and efficacious intervention that offers clinically meaningful improvements in cardiorespiratory fitness and body composition for colorectal cancer survivors	[83, 103]

## Exercise during treatment

Surgical process and chemotherapy are two main therapeutic strategies in treating CRC patients. Various studies have shown that exercise and physical activity are effective in increasing patients' tolerance and decreasing the side effects of these modalities [85]. In the case of the effects of an exercise intervention on patients' quality of life before or after surgery, there is a limited number of completed studies and ongoing clinical trials. In a randomized controlled trial study by Ahh et al., it was reported that post-operative exercise consisted mostly of stretching and very-low-intensity resistance exercises effectively decreased length of hospital stay and improve bowel motility after a surgical procedure in patients with stages I–III CRC [104]. A clinical trial aimed to investigate the effects of a training program with intensified physical activity before and after a surgical procedure on surgical-related postoperative recovery time, hospital stay, sick leave, and complication rate, is ongoing [105]. In patients undergoing chemotherapy, an 18-week supervised exercise program in 33 CRC patients, was shown to be safe and feasible. The intervention significantly reduced physical fatigue at 18 weeks and general fatigue at 36 weeks [106]. In addition, an aerobic exercise program for 150 min or more per week for 6–8 weeks during and after neoadjuvant chemo-radiotherapy (NACRT) is also safe in CRC patients [107]. Similar results were reported for the training performed three times per week for 1 year in 30 CRC patients [108]. A combined aerobic and resistance exercise program was shown to improve muscle strength, cardiorespiratory fitness, emotional distress, physical activity, fatigue, and sleep quality in patients with stage II-III CRC patients undergoing chemotherapy [109]. Chemotherapy-induced side effects such as peripheral neuropathy, fatigue, muscle weakness, pain, cardiovascular and pulmonary complications, immune dysfunction, anemia, anxiety, depression, sleep disorders, and endocrine changes are also alleviated by exercised training in CRC patients [57, 110, 111].

## Molecular mechanisms

**Fig. 2:**
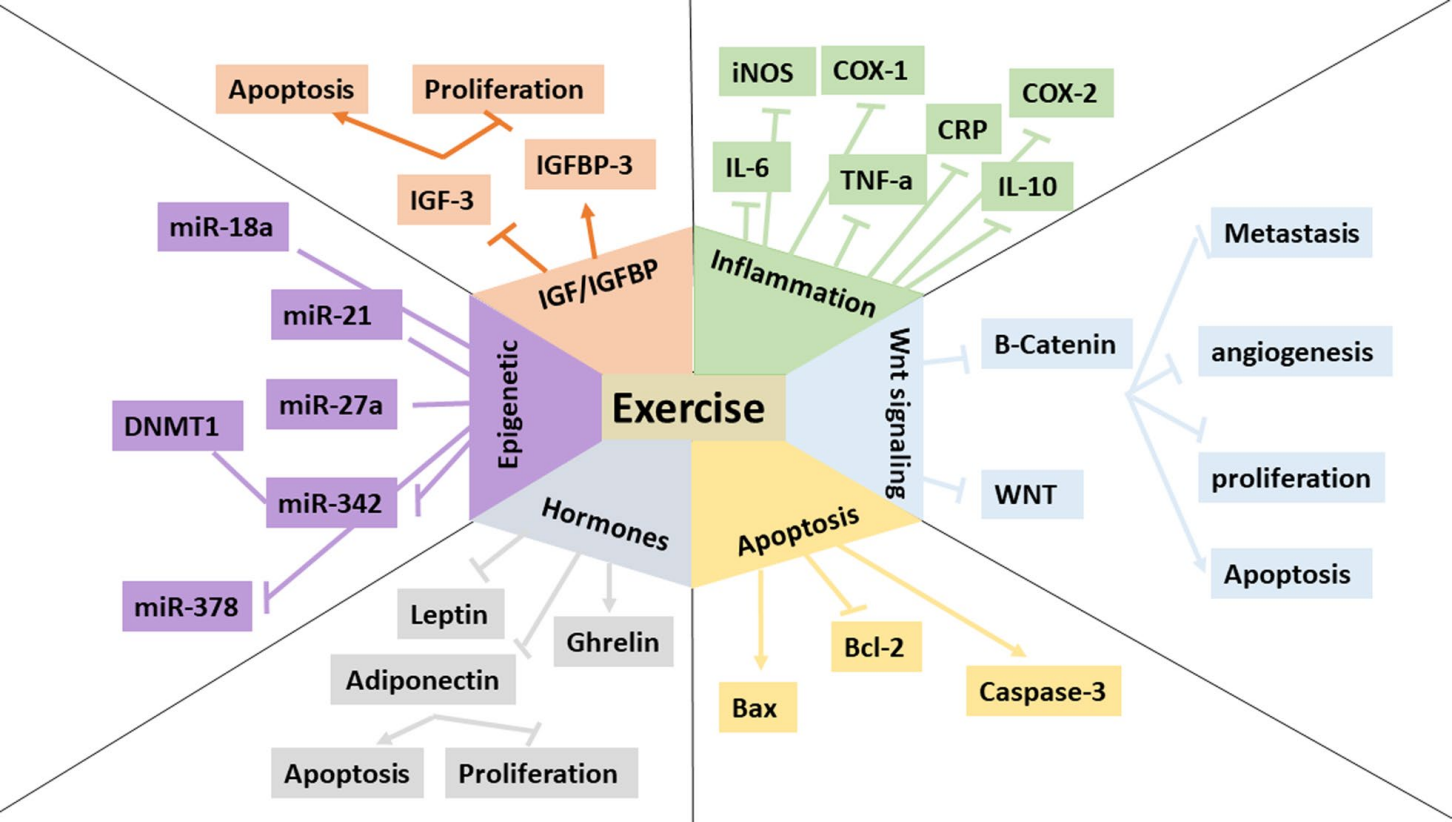
molecular mechanisms of preventive effects of exercise in colorectal cancer

Recent years have witnessed a huge increase in the number of clinical trials focusing on the efficacy of various types of exercise programs in the treatment of CRC. Table 3 shows a long list of clinical trials extracted from clinicaltrials.gov. However, there is a limited number of studies investigated the mechanisms underlying the positive effects of physical activity in CRC. Previous reviews have focused on the beneficial therapeutic and preventive effects of exercise in CRC, however, there is not a comprehensive review focusing on the molecular mechanisms. Therefore, these mechanisms are not still fully understood and need more basic and deep investigations. In the next section of the present review, we will discuss some important and well-studied mechanisms, which are suggested to be the lost pieces in the puzzle of physical activity and CRC (Fig. 2).

**Table 3:** Clinical trials investigating positive roles of exercise training in colorectal cancer

Cancer type	Sample size	Exercise intervention	Goals	status	number
Colon Cancer Rectal Cancer	134 patients who have completed treatment for colorectal cancer in the past 2 years 18 Years and older	Moderate-intensity physical activity	The efficacy of the physical activity intervention on fitness, vigor, fatigue, physical functioning, and body esteem	Completed	NCT00230646
Colorectal cancer	207 participants 50 Years to 80 Years	Participants will read 10 behavioral messages about colon cancer screening and physical activity	Design messages which can help people learn more about how to prevent colon cancer	Completed	NCT00924690
Colorectal Cancer Obesity	40 participants 50 Years and older Body mass index 25–40 kg/m^2^	Physical Activity and Energy Restriction	Studying diet and physical activity in healthy overweight, obese, or inactive participants at risk of developing colorectal cancer	Completed	NCT00653484
Colorectal Cancer	202 participants 40 Years to 75 Years	60 min/session of aerobic exercise, 6 d/week at 50–50% HHR. All sessions will begin with 10 min of stretching and 5 min of warm-up, and will end with 5 min of cool down	Investigate the efficacy of a one-year moderate intensity aerobic exercise program in modulating these processes to a pattern considered low risk for colon cancer Investigate the mechanisms whereby exercise may lower colon cancer risk in humans	Completed	NCT00668161
Colorectal Cancer	39 participants 18 Years and older	Patient allocated to the InterWalk-group will be introduced to the InterWalk app, including instructions on how to down load and use the application. and prescribed to perform Interval Walking for 150 min per week	Investigate interval-walking, delivered by the InterWalk smart phone application as exercise-modality in patients with colorectal cancer	Completed	NCT02403024
Colorectal Cancer	36 participants 60 Years and older	The intervention is a workbook that coaches participants through walking regularly at a safe, comfortable pace with the ultimate goal of walking at least 30 min a day five days a week	Evaluate the effects of physical activity intervention on fatigue and quality of life during chemotherapy for CRC patients	Completed	NCT02191969
Colorectal Cancer Depression Fatigue Pain	50 participants up to 120 Years	150 min/wk of moderate exercise through use of the Curves® centers, engage in physical activity outside of Curves®, and use pedometers to track activity	Studies how well exercise, diet, and counseling work in improving physical activity and weight loss in overweight women who are breast and colorectal cancer survivors	Completed	NCT01453452
Anxiety Disorder Colorectal Cancer Depression	45 participants 18 Years and older	Moderate Physical Activity	Studying how well physical activity helps patients with stage II or stage III colorectal cancer recover from cancer	Completed	NCT00373022
Colorectal Cancer	50 participants 40 Years to 80 Years	The patients will perform increasing volumes of moderate intensity endurance (e.g. walking, cycling) exercise, leading up to 18 MET-hours per week by the end of three months. Patients will then maintain this activity level for the remaining 9 months, with reduced supervision	to determine the feasibility of a one year exercise training program in post-surgical patients with colorectal cancer	Completed	NCT01991847
Colorectal Carcinoma	11 participants 70 Years and older	Participants will complete the High-Intensity Functional Exercise (HIFE) programme. The programme improves lower-limb strength, balance and mobility and all the exercises can be performed by the individual at home and with minimal equipment	The Assessment of the Feasibility of a Home Based Exercise Programme in the Older Patient Following Major Surgery	Completed	NCT03064308
Colorectal Carcinoma	16 participants 18 Years and older	The exercise regime consists of 10 sessions of exercise against a constant load, each lasting 60 s, separated by 60 s of recovery eliciting 90% maximal heart rate. The 6 sessions are held over a 2 week period	To determine clinical and biological effects of a preoperative exercise programme in colorectal tumour and skeletal muscle tissues	Completed	NCT02056691
Colorectal Cancer	50 participants 18 Years and older	motivational counseling to exercise a minimum 18 metabolic hours per week	To evaluate compliance at 6 months with post-treatment recommendations for a minimum of 18 metabolic units of physical activity each week in patients who have completed therapy for stage 2 and stage 3 colorectal cancer	Completed	NCT00977613
Colorectal Cancer	24 participants 18 Years and older	A 2–4 week low volume, moderate intensity, supervised, one to one, individualised exercise programme	To determine whether a defined exercise programme can improve recovery and reduce complications after surgery	Completed	NCT02264496
Colorectal Cancer	44 participants 18 Years and older	Aerobic exercise	To identify the dose–response effects of aerobic exercise on molecular and cellular pathways associated with physical activity and CC outcomes among patients with stage II and III CRC	Completed	NCT02250053
Colorectal Cancer	788 participants 18 Years and older	12 supervised exercise sessions over 3 months and six supportive behavior change workshops	Investigating the feasibility of a behavior change intervention based on Self-Determination Theory in people recovering from colorectal cancer and its effects on behavior change 6 months post-intervention	Completed	NCT02751892
Colorectal Cancer	52 participants 18 Years and older	The exercise group will perform supervised stationary cycle ergometer exercise 3 times per week for 12 weeks and be progressed from 15 to 45 min and 60% to 110% of the power output obtained at V02peak. Resistance training will be completed twice per week and will include exercises for all major muscle groups. The training will progress from 60 to 80% of 1RM over the course of the intervention	investigate hypothesize that an exercise training program will be a safe, feasible, and effective intervention to improve the fitness and body composition of a group of colon cancer survivors	Completed	NCT00813540
Colorectal Cancer	35 participants 18 Years and older	Exercise Training	Does Short-term Exercise Intervention Improve Pre-operative Physical Fitness Following Neoadjuvant Chemoradiotherapy in Colorectal Cancer Patients?	Completed	NCT01325909
Bladder Cancer Breast Cancer Colorectal Cancer Esophageal Cancer Fatigue Lung Cancer Lymphoma Ovarian Cancer Prostate Cancer	49 participants 60 Years and older	Pelvic floor muscle training for one month (2 ~ 3 times a week, for 4 weeks) just one month before the stoma closure	Patients undergo resistance exercise via negative-eccentric work (RENEW), using a special seated stationary leg exercise machine, 3 times a week for up to 12 weeks	Completed	NCT00335491
Colorectal Cancer	27 participants 21 Years and older	International physical activity questionnaire	To quantitatively assess the average amount of physical activity that patients are capable to perform while receiving regorafenib for the treatment of metastatic colorectal cancer	Completed	NCT02347852
Colorectal Cancer	35 participants 18 Years and older	Yoga	to determine if individuals with colorectal cancer enjoy yoga and to begin to assess whether yoga is effective in improving attention and immune function in individuals with colorectal cancer compared to physical activity and usual care	Completed	NCT02564835
Colon Cancer Rectal Cancer Frailty	48 participants 18 Years and older	A minimum of four weeks of prehabilitation with exercise three times a week, protein and vitamin supplements, dietitian consultation and medical optimization prior to surgery	Effects of Multimodal Prehabilitation in Colorectal Cancer Patients	Recruiting	NCT04167436
Colorectal Cancer Depression Fatigue Psychosocial Effects of Cancer and Its Treatment Sleep Disorders	962 participants 18 Years and older	3 phases Phase 1: Intensive intervention for 6 months Phase 2: Reduced intervention for months 6–12 Phase 3: Minimal intervention for months 12–36	This randomized phase III trial is studying a physical activity program given together with health education materials to see how well it works compared with giving health education materials alone for patients who have undergone treatment for high-risk stage II or stage III colon cancer	Recruiting	NCT00819208
Colorectal Cancer Surgery	500 participants Child, Adult, Older Adult	Exercise therapy during at least 2 weeks	whether prehabilitation is cost-effective in colorectal cancer surgery among individual patients aged 70 years and above or patients with an American Society of Anesthesiologists (ASA) score of III. We also aim to identify factors facilitating or impairing implementation of prehabilitation such that it is cost-effective	Recruiting	NCT04097795
Colorectal Cancer	30 participants 19 Years to 75 Years	Participants will be instructed to complete four, high-intensity interval training workouts per week at home, for the duration of the 12-week trial	provide us with preliminary evidence for a larger trial aimed to compare the effectiveness of these two different types of home-based exercise programs on physical outcomes linked with survival, quality of life, and surrogate blood markers of colorectal cancer recurrence	Recruiting	NCT04080414
Colorectal Cancer	70 participants 18 Years and older	Trimodal prehabilitation application in the form of control with the 6-min walking test and podometer of physical activity	To investigate the effects of physical activity on morbidity and mortality and in addition to postoperative recovery	Recruiting	NCT03543514
Colorectal Cancer	80 participants 18 Years and older	Physical activity	how the physical activity level before operation of colon cancer affects the outcome of complication and histology	Recruiting	NCT03947840
Colorectal Cancer	15 participants 50 Years to 90 Years	30-min of moderate-intensity aerobic exercise	evaluate whether adding the exercise serum to colon cancer cells in a dish can reduce the growth of the cells compared to the resting serum	Recruiting	NCT04057274
Colorectal Cancer	300 participants 18 Years to 80 Years	12-week structured physical activity program	to assess whether a structured physical activity program (PA) during palliative chemotherapy improves progression-free survival (PFS) and/or patient-reported outcomes (ESAS-r) in patients with metastatic colorectal cancer	Recruiting	NCT02597075
Colorectal Cancer	300 participants 18 Years and older	Endurance exercise	investigate the efficacy of endurance exercise following adjuvant chemotherapy in patients with colorectal cancer	Recruiting	NCT03822572
Colorectal Cancer	72 participants 18 Years and older	Graduated walking programme, strengthening exercises and respiratory muscle training	the effect of home-based prehabilitation on the cardiorespiratory fitness of high-risk colorectal cancer patients awaiting surgery	Active, not recruiting	NCT03336229
Colorectal Cancer Rectal Cancer	48 CRC patients on chemotherapy 18 Years and older	Physical activity tracker wristband and daily text messages delivered to the participants' phones	Determine the feasibility of the intervention via adherence and attrition, and determine the acceptability of the intervention via questionnaires and semi-structured interviews Estimate the effect of the intervention vs. usual care on physical activity, QOL, and symptoms at 12-weeks Explore the impact of the intervention vs. usual care on fitness, weight, waist circumference, and blood pressure at 12-weeks	Active, not recruiting	NCT03524716

### Inflammation

An accumulating number of evidence demonstrates that inflammation has a broader range of effects on CRC pathogenesis, from supporting primary tumor growth by promoting tumor cell proliferation to helping angiogenesis by increasing the availability of pro-angiogenic molecules, to suppressing anti-tumor immunity by recruiting anti-inflammatory cell types, and to shaping pre-metastatic niches to promote subsequent metastasis. In addition to the critical role of inflammation and inflammatory mediators in the initiation/ progression of CRC, it is suggested that this process has also been involved in the preventive effects of exercise on CRC [112]. Physical activity is demonstrated to have an inhibitory effect on systemic inflammation by decreasing various pro-inflammatory cytokines such as interleukins, C-reactive protein, and tumor necrosis factor (TNF)-α [113]. In animal models of CRC, numerous studies have evaluated the roles of exercise in the suppression of inflammatory events. For example, Mehl et al. [114] reported that treadmill running significantly decreased plasma IL-6 levels in APC^min/+^ male mice, hence inhibit CRC progression, as was shown by the decreased number of polyps. In a study by Frajacomo et al. [115] it was demonstrated that interleukin (IL)-10 was a vital element for anti- preneoplastic effects of aerobic training on the colon. They showed that aerobic training mice developed 36% less colon preneoplastic lesions than their controls. However, Knocking IL-10 out mice abrogated the anti-preneoplastic effects of aerobic training on the colon tissue [115]. Darband et al. [116] showed that exercise on a treadmill 5 days/week for 8 weeks reduced ACF. They reported that suppressing inflammation was a major underlying mechanism since serum levels of IL-6 and TNF-α, and expression levels of cyclooxygenase (COX)-1 in colon tissue were significantly elevated in the rats receiving 1, 2-dimethylhydrazine (DMH) and downregulated after performing the exercise program. COX enzymes (COX-1 and -2), which have key functions in intestinal tumor formation, are also indicated to be mediators of exercise beneficial effects on CRC [116]. Demarzo et al. [117] reported that swimming training resulted in the decreasing number of ACF in rats with DMH- induced CRC through the downregulation of COX-2. Exercise also resulted in decreasing local inflammation by decreasing inducible nitric oxide synthase (iNOS) expression in the colon mucosa in azoxymethane (AOM)-induced CRC in mice [117]. In a study by Baltgalvis et al. the effects of regular moderate-intensity treadmill exercise training in attenuating polyp formation in Apc^(Min/+)^ mice fed the Western-style die were investigated. The authors found that exercise reduced total intestinal polyp number by 50% and the number of large polyps by improving the markers of systemic inflammation and immune system function. The Western-style diet increased polyp number by 75% when compared with control mice, but exercise did not decrease polyp number or alter polyp size in mice fed the Western-style diet. These data suggest that the induction of adiposity, inflammation, and immunosuppression by the Western-style diet may compromise the beneficial effect of moderate-intensity exercise on the intestinal polyp burden in Apc^(Min/+)^ mice [118].

### Insulin-like growth factor axis

The insulin-like growth factor (IGF) system plays a pivotal role in the pathogenesis, progression, and prognosis of CRC. Previous evidence indicates that hyperactivation of the IGF pathway represents an early step in colon cancerogenesis, establishing both mitogenic and pro-angiogenic signals that favor neoplastic transformation of normal colorectal epithelial cells [119]. The IGF axis is one of the most substantial mechanisms with well-defined roles in exercise activity and CRC [120]. Due to the main functions of IGF in the regulation of key cellular processes such as proliferation, differentiation, and apoptosis, these proteins and their binding proteins (IGFBPs) are a hot point in researching about CRC pathogenesis [120]. IGF-1 upregulation is reported to be linked to CRC risk [121]. The importance of the IGF axis in CRC incidence, initiation, and progression is strongly supported by observational and preclinical studies [122–127]. As a result, exercise-mediated manipulation of the IGF axis is considered as a preventive therapy for CRC, which may be effective in decreasing CRC-specific mortality. In general, there is an inconsistency in the physiological response of IGFs to physical activity [128–133]. In other words, different studies have reported different responses of the IGF axis to exercise, which is proposed that relates to negative energy balance, physical, conditioning and energy flux [128, 129]. The six-week voluntary exercise was shown to decrease the ratio of serum IGF-1 to IGFBP-3 levels, hence inhibit intestinal tumorgenesis in Apc^Min/+^ mice. It was suggested that the inhibitory role of exercise on colon carcinogenesis is related to decreased IGF-1/IGFBP-3 ratio [134]. In addition, 8-week resistance training was also demonstrated to reduce serum IGF-1 level and IGF-1/IGFBP-3 ratio in rats, which is considered as a link between resistance training and lower risk of CRC [135]. Investigating 526 CRC survivors have demonstrated that for the physically active patients, increasing IGFBP-3 by 26.2 nmol/l was associated with a 48% reduction in CRC specific deaths [136]. In an interventional study by Lee et al. it was reported that a 12-week home-based exercise program resulted in a significant reduction in insulin and IGF-1 levels, as well as an increase in IGFBP-3 levels in 70 patients with stage II–III CRC survivors [137]. Therefore, heterogeneous results decreased IGF-1 levels, and increased IGFBP-3 levels may be a reasonable mechanism underlying the inverse correlation between CRC and physical activity [120]. The association between exercise and CRC cannot be explained by using a single mechanism because exercise and interrelated factors exert varying effects.

### Immunity

Exercise is shown to be a major modulator of the immune system. However, the exact role of this interaction is not yet completely understood. On the other hand, the key players of the immune system, including T cells and macrophages have also been demonstrated to play a critical function in CRC pathogenesis, such that an increased number of these immune cells is associated with poor prognosis in CRC patients [138, 139]. In a study by McClellan et al. [140] it was reported that treadmill running for 1 h/day and 6 days a week at 15 m/min resulted in the decreased expression levels of specific markers for macrophage (IL-12, IL-23 and Nos2, CD206, IL-10, IL-4, CCL17, CCL22, and Arg-1) and T-cells (CD8 and Foxp3), hence led to reduced CRC progression. Other studies also confirmed the modulatory effects of exercise and physical training on the immune system in animal models [141, 142].

### Epigenetics and miRNA

The interaction between physical activity and epigenetics is based on the evaluation of variation in patterns of DNA methylation at CpG sites within specific genes with particular biological roles [143–145]. Molecular epidemiology has identified various target genes including adenomatous polyposis coli (APC), MutL homologue 1 (MLH1), tumor growth factor beta (TGF-β), the cyclin-dependent kinase inhibitor p16, K-Ras (KRAS), and B-Raf (BRAF), that are differentially methylated in normal versus neoplastic colonic epithelium [146, 147]. In other words, increased methylation in mentioned genes are frequently observed in CRC tissues, which suggests the critical function of these genes and their functional proteins in the pathogenesis of CRC. With regard to CRC, a limited number of studies have evaluated the interaction between physical activity and DNA methylation. In spite of the informative nature of these studies, due to some limitations, all studies did not find a significant association between exercise and DNA methylation at promoters of IGFBP, MLH1, BRAF genes, and p15 tumor suppressor gene [148–151]. In addition to DNA methylation, the effects of exercise on the expression levels of microRNAs (miRNAs) were also investigated in some recent studies. In a study by Tonevitsky et al. it was reported that a 30 min of exercise had a direct effect on the expression levels of miR-21, miR-27a, and miR-18a eight adult males, all of which are involved in CRC pathogenesis [152]. In another study, it was found that exercise resulted in the decreased expression levels of miR-342, which targeted the DNA methyltransferase gene (DNMT1) [153]. In rats with Azoxymethane-induced CRC, Kriska et al. [154] showed that the colon, muscle, and serum expressed miR-378 inversely proportional to CRC progression; and treatment with a 24-week progressive treadmill-training program (1 h/d, 3 d/wk) resulted in suppression of cancer progression and increase in miR-378 expression.

### Leptin and ghrelin

Leptin and ghrelin are two regulators of energy balance and weight control. Physical activity is also contributed to the regulation of the expression levels of these two hormones. Exercise increases ghrelin levels and decreases leptin levels [155, 156]. On the other hand, CRC cells exposure to adipocytes and pre-adipocytes has been found to increase cellular proliferation [157, 158]. In a study by Nuri et al. [159] the exercise program consisted of 8 weeks walking and three 45 min sessions in each week with 50–60% of the target heart rate in 30 men with CRC resulted in increased ghrelin levels; however, plasma leptin and insulin resistance were not affected by this protocol in male patients with CRC. In another study by Piringer et al. [108] exercise 3 times per week for 1 year resulted in significant increases in adiponectin and leptin levels in 30 CRC patients.

### Oxidative stress

Perturbance in the oxidative balances is suggested as one the main mechanisms involved in the development of colorectal cancer [6]. Exercise-mediated suppression of oxidative stress, though, is a considered as a therapeutic mechanism in CRC. For example, Perse et al. demonstrated that exercise exerted protective effects on developing CRC is induction of oxidative stress. However, in terms of the combined effects of dietary fat and exercise, they indicated that the protective role of exercise was significantly depressed by a high fat mixed lipid (HFML) diet. An HFML diet significantly reduced the protective influence of exercise on colon carcinogenesis in rats and affected the degree of peroxidation in the large bowel during exercise, as well as concentrations of serum enzymes (LDH, α-HBDH, CK, ALT and AST) [160]. The authors, in another study, reported that endurance swimming prevented lipid peroxidation in the soleus muscle of HFML diet rats due to elevated activities of antioxidant enzymes. On the other hand, increased lipid peroxidation in the hearts of all cancer groups indicated that DMH-induced colon carcinoma impaired the antioxidant status of the heart. This failure in heart tissue indicated that enhanced anti-oxidant capacity after regular physical activity is not sufficient to offset oxidative stress caused by DMH-induced colon carcinoma [160]. Therefore, exercise exerted a protective effect in developing CRC via increasing the antioxidant capacity.

### Apoptosis

It is now well-established that dysfunction in apoptotic pathways, which plays a pivotal function in maintaining tissue homeostasis, is one of the main contributors to tumorigenesis [161]. However, there is very little data on the benefits of exercise on apoptosis in the settings of CRC. Darband et al. [116] reported that an 8-week moderate-intensity exercise program resulted in the decreased number of aberrant crypt foci (ACF) and improvement in colon architecture in rats with DMH- induced CRC. They found that exercise upregulated apoptosis, which was evident from the increased Bax/Bcl2 ratio, and enhanced the expression levels of activated caspase-3 as compared to the DMH group [116]. In another study, it was shown that moderate-intensity exercise also modulated apoptosis in Apc^Min/+^ mice, hence led to a 35% decreased in colon polyp formation and growth. In addition, exercise downregulated the expression levels of Bax in the colon tissue of Apc^Min/+^ mice [162]. Therefore, exercise is a major regulator of apoptosis and its components. However, research in this area is in its infancy and needs more investigations.

### Signaling pathways

The Wnt/β-catenin signaling pathways are one the most important signaling involved in the initiation/progression of CRC, with major roles in cellular proliferation, apoptosis, angiogenesis, and metastasis [31, 163, 164]. Loss of APC and its major mediator CTNNB1 (β-catenin), which is a common event in the early stages of CRC, leads to an increase in cellular proliferation independent of the energy balance [165]. There is a mutual interaction between exercise and Wnt/β-catenin signaling, such that exercise changes the WNT-CTNNB1 signal in colonic mucosa and the WNT-CTNNB1 pathway modulates the cellular sensitivity to exercise [134]. In animal models, exercise resulted in an increase in the phosphorylation of β-catenin in the colon tissue of mice with CRC [162]. Morikawa et al. demonstrated that in the active early-stage CTNNB1 negative CRC patients, who performed ≥ 18 MET hour/week of exercise after CRC diagnosis, the incidence rate of CRC-specific mortality decreased by 67%, in comparison to inactive patients [166]. In another study, it was found that patients with weak staining for β-catenin in the exercise program had a lower mortality rate [167]. Therefore, CTNNB1 status can be used as a predictive biomarker in response to exercise applications [168].

## Future directions

There are several gaps in evidence identified in this review that deserve attention. First, few studies have been designed with the goal of investigating the therapeutic efficacy of exercise in CRC, as well as improving treatment efficacy. Second, very few studies included radiotherapy, immunotherapy, or other more recently developed anticancer therapies, which may also interact with exercise. Third, the number of studies investigating the mechanisms underlying exercise mediated protective effects, particularly downstream signaling pathways is very low. Despite the inherent limitations of the reviewed studies, the evidence presented here is promising. In the past four decades, over 700 exercise trials in the oncology setting and more than 30 trials in colorectal cancer have been conducted to establish evidence for safety and feasibility, and whether exercise can improve physical function and quality of life outcomes among cancer survivors. Clinical studies with treatment efficacy as the primary outcome have been far fewer, probably because of the necessity of longer follow-up larg, er sample sizes, and limited funding opportunities. Nevertheless, treatment efficacy is the most important issue for CRC patient, and we need to pay more attention to this area. Existing and future cohort studies that collect physical activity information before or during cancer treatment should consider the feasibility of data linkage in their design to examine the associations between physical activity and cancer treatment outcomes. These studies may also begin to examine the biological mechanisms underlying the relationship between physical activity and cancer treatment efficacy by including biological samples (i.e., markers of angiogenesis, immune function, inflammation, metabolism). Alternatively, ongoing efficacy trials of new cancer treatments may collect physical activity information to examine their influence on treatment efficacy outcomes.

## Conclusion

The health beneficial effects of exercise and physical activity have been proven and considered for many years and recent decades are witnessed with an increased number of studies investigating the effects of exercise and physical activity in preventing and treating various human malignancies, including CRC. In this regard, an accumulating number of observational and experimental studies have shown the modulatory function of exercise on CRC initiation/progression. Based on these studies, physical activity and avoiding a sedentary lifestyle have major positive effects in decreasing CRC incidence and mortality. In addition, applying exercise is also effective in improving the quality of life in CRC survivors and the severe side effects of various therapeutic strategies in CRC patients. Furthermore, various studies have introduced different underlying mechanisms for the beneficial effects of the exercise and physical activity in CRC, some important of them, which was discussed in this review, include suppression of inflammation, modulation signaling pathways such as IGF axis and β-catenin, and regulation of apoptosis and immunity. Investigating exercise targeting mechanisms is CRC is still in its infancy and needs further studies for better understanding the nature of exercise in CRC and identifying appropriate biomarkers for CRC.

## Data Availability

Not applicable.
